# Higher Serum CCN3 Is Associated with Disease Activity and Inflammatory Markers in Rheumatoid Arthritis

**DOI:** 10.1155/2020/3891425

**Published:** 2020-05-09

**Authors:** Yingying Wei, Linan Peng, Yi Li, Na Zhang, Ke Shang, Lihua Duan, Jixin Zhong, Jie Chen

**Affiliations:** ^1^Department of Rheumatology and Clinical Immunology, Jiangxi Provincial People's Hospital, Nanchang University, Nanchang, China; ^2^School of Medicine, Xiamen University, Xiamen, China; ^3^Department of Rheumatology and Immunology, Tongji Hospital, Tongji Medical College, Huazhong University of Science and Technology, Wuhan, Hubei, China; ^4^Department of Scientific Research and Education, Jiangxi Provincial People's Hospital, Nanchang University, Nanchang, China

## Abstract

Nephroblastoma overexpressed protein (NOV/CCN3), the early discovered member of the CCN family, has recently been suggested to be involved in a number of inflammatory processes, including wound healing, alveolar epithelial cell inflammation, cancer metastasis, and macrophage foam cell formation. However, the role of CCN3 in rheumatoid arthritis (RA), a classic autoimmune and inflammatory disease, remains elusive. RA is a chronic systemic autoimmune disease that eventually leads to cartilage and bone destruction and joint dysfunction. In this study, we investigated the potential of serum CCN3 as a biomarker for RA. The serum levels of CCN3 were measured by ELISA. The clinical and laboratory parameters were collected from a clinical record system, and disease activity was determined by joint disease activity score 28 (DAS28). Our results showed that the serum levels of CCN3 were significantly increased in RA patients compared to healthy controls. Furthermore, the CCN3 level was positively correlated with DAS28 (CRP), DAS28 (ESR), and the level of anti-CCP Ab, an autoantibody highly specific for RA. Furthermore, CCN3 showed a positive correlation with inflammatory cytokine IL-6, while no significant correlation with TNF-*α* was observed. These data suggest that CCN3 plays an important role in the development of RA and might be a potential disease activity biomarker for RA.

## 1. Introduction

RA is a chronic and systemic autoimmune disease with pathological characteristics of sustained inflammatory synovitis, pannus formation, and abundant lymphocyte infiltration, followed by the destruction of joint cartilage and bone. A number of risk factors are involved in the development of RA, including infection, sex hormones, and genetic background [1, 2]. For example, it is reported that HLA-DRB1 mutation is associated with an increased risk of RA [3]. Recent studies have demonstrated that immune imbalance plays a key role in the pathogenesis of RA. TNF-*α* and IFN-*γ*, which are Th1-related cytokines, are considered as the key proinflammatory cytokines in the progression of human RA [4, 5]. In contrast, most studies have shown that Th2-type immune response alleviates Th1-mediated RA [6]. Recently, the imbalance of Th17 and regulatory T cell (Treg) has also been shown to play a critical role in autoimmune diseases [7], including human RA and collagen-induced arthritis [7, 8].

Nephroblastoma overexpressed protein (NOV/CCN3) is a member of the CCN family, which includes CYR61/CCN1, CTGF/CCN2, NOV/CCN3, WISP1/CCN4, WISP2/CCN5, and WISP3/CCN6 [9]. CCN3 gene was firstly identified as an integration site from nephroblastoma of newborn chicken infected with myeloblastoma-associated virus (MAV) [10]. Previous studies have demonstrated that CCN3 protein is involved in multiple biological activities including cell adhesion, migration, and proliferation. Furthermore, CCN3 can be released out of cytoplasm and serves as a matrix molecule in a number of pathophysiological processes such as wound healing, angiogenesis, and fibrosis [10–13]. In addition, CCN3 also regulates the expression of inflammatory molecules in astrocyte and promotes the regeneration of central nerve system (CNS) myelin. A recent study suggested that CCN3 could be detected in RA and OA synovial tissues [14]. CCN3 knockout mice displayed osteoarthritic changes in knee articular cartilage, suggesting that CCN3 may suppress osteoarthritis progression by maintaining the differentiated phenotype of articular cartilage [15]. However, the role of CCN3 in RA remains unknown.

In the present study, we examined the serum levels of CCN3 by ELISA in RA patients. The correlations with clinical disease activity and inflammatory markers were analyzed. We found that the serum levels of CCN3 were significantly higher in RA patients compared to those in healthy subjects. Particularly, the CCN3 level was positively correlated with the disease activity score. Previous studies showed that the anti-cyclic citrullinated peptide (CCP) antibody is an autoantibody highly specific for RA and is predictive of radiological involvement in RA patients. In this study, we found that CCN3 expressions of RA patients were positively correlated with the serum level of anti-CCP antibody and RA disease activity score. Furthermore, a positive correlation between CCN3 and IL-6 was also observed in RA patients. These data suggest that CCN3 may be an inflammatory factor during the process of RA and may serve as a biomarker or an alternative therapeutic target in managing RA.

## 2. Materials and Methods

### 2.1. Patients and Control Subjects

A total of 41 RA patients were recruited from the Department of Rheumatology and Clinical Immunology, Jiangxi Provincial People's Hospital Affiliated to Nanchang University. The RA patients were all diagnosed complying with the 1987 revised diagnostic criteria of the American College of Rheumatology (ACR) [16]. Patients with various types of arthritis including osteoarthritis (OA), septic arthritis, psoriatic arthritis, and reactive arthritis were excluded. We recruited 45 age- and sex-matched healthy volunteers as the control. Informed consent of all the participants and the approval of the medical ethics committee of the hospital were obtained in accordance with the regulation.

### 2.2. Routine Laboratory Parameters

The clinical and laboratory parameters of RA patients were gathered from the clinical record system. The disease activity parameters were represented by disease activity score 28 (DAS28) (ESR) and DAS28 (CRP) as described previously [17].

### 2.3. Detection of Cytokines by ELISA

The peripheral blood samples of RA patients and healthy controls were collected into sterile coagulant tubes. Sera were isolated by centrifugation at 3500 rpm for 5 min and then kept frozen at -80°C. Concentrations of CCN3, IL-6, and TNF-*α* (R&D, Minneapolis, MN) were measured by ELISA kits according to the manufacturer's manuals.

### 2.4. Immunohistochemical Analysis

The anti-CCN3 antibody was purchased from R&D (Minneapolis, MN). After deparaffinization and rehydration, the sections were treated with 3% H_2_O_2_ followed by blocking with 10% goat serum in PBS. Then, the sections were stained with anti-CCN3 antibody overnight at 4°C, and afterwards, a hypersensitive two-step immunohistochemical detection reagent (ZSGB-BIO, China) was applied to detect the CCN3 expression under a microscope.

### 2.5. Statistical Analysis

GraphPad Prism 5 was used for statistical analysis. The Whitney *U* test was applied to analyze the difference between RA patients and healthy controls. Data were expressed as the mean ± standard deviation (*M* ± SD). The Spearmen test was adopted for correlation analysis, and *p* < 0.05 was considered statistically significant.

## 3. Results

### 3.1. Clinical Characteristics of RA Patients

The clinical characteristics of RA patients were summarized in Table 1. Forty-one patients with RA and forty-five healthy controls were enrolled. The mean age for RA patients was 51.7 years with an age range from 26 to 71, and there were 36 females and 5 males. No significant differences in age and sex were observed between RA patients and healthy controls. The mean of disease duration was 10.6 years with a range of 0.5–20 years. As expected, ESR, the serum levels of C-reactive protein (CRP), rheumatoid factor (RF), and anti-CCP antibody were markedly higher in RA patients than those in healthy controls (Table 1).

### 3.2. Increased Serum CCN3 Level in RA Patient

**Figure 1:**
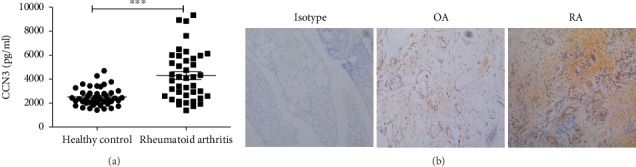
Increased CCN3 expression in RA patients. (a) The sera isolated from rheumatoid arthritis patients (RA, *n* = 41) and healthy controls (HC, *n* = 45) were used for the detection of CCN3 by ELISA. The Mann-Whitney *U* test was conducted to compare the data between two groups. ∗∗∗ indicates *p* < 0.001. (b) Local expression of CCN3 in joint tissues collected from patients with OA or RA. The expression was examined by immunohistological analysis (magnification 100x).

It has been demonstrated that CCN3 plays a critical role in many diseases, such as central nervous system and cardiovascular diseases [18–20]. However, the role of CCN3 in the development of RA has not been described. To investigate whether CCN3 is involved in the development of RA, the sera of RA patients and healthy controls were collected, and the serum CCN3 level was measured by ELISA. As shown in Figure 1(a), the serum CCN3 level in RA patients was significantly higher compared with that in controls (*p* < 0.0001). The mean level of CCN3 in RA patients was 4288 pg/ml with a range of 1395-9233 pg/ml, while the healthy control was 2506 pg/ml (1409-4691 pg/ml). In addition, the deposition of the CCN3 in paraffin-embedded joint tissues was also determined. We found a considerable deposition of CCN3 in the joint tissues from RA patients, but not in the control tissues collected from OA patients (Figure 1(b)).

### 3.3. Serum CCN3 Level and Its Correlation with Disease Activity in RA Patients

**Figure 2:**
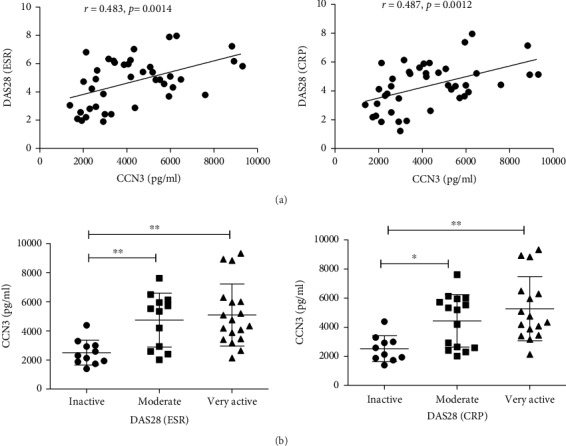
Association of CCN3 with disease activity in RA patients. (a) The determination of linear relationship between serum CCN3 expression and DAS28 in RA patients was performed by the Spearman correlation coefficient. (b) The RA patients were divided into inactive, moderate, and very active groups by the DAS28 (ESR) and DAS28 (CRP). Serum CCN3 levels were compared among these groups, and the difference was evaluated by the Mann-Whitney *U* test. ∗ indicates *p* < 0.05; ∗∗ indicates *p* < 0.01.

Previous studies have shown that CCN3 plays an important role in many diseases, including inflammatory diseases [20, 21]. As RA is an inflammatory disease and the expression of CCN3 was highly increased in RA patients, the correlation between CCN3 and disease activity score was analyzed. As expected, the serum CCN3 level was positively correlated with DAS28 (ESR) (*r* = 0.483, *p* = 0.0014) and DAS28 (CRP) (*r* = 0.487, *p* = 0.0012) (Figure 2(a)). We further divided the RA patients into inactive, moderate, and very active groups, according to the DAS28. We found that the patients with a higher disease activity score showed a higher CCN3 level when compared with inactive patients. The serum CCN3 was 2519 pg/ml (1385-4691 pg/ml) in inactive patients (DAS28 (CRP)), 4429 pg/ml (2015-7603 pg/ml) in moderate patients, and 5262 pg/ml (2131-9322 pg/ml) in active patients (Figure 2(b)).

### 3.4. Association of Serum CCN3 Level with Laboratory Parameters

**Figure 3:**
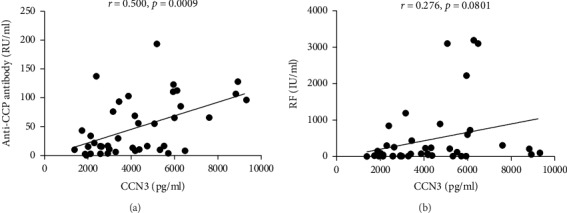
Positive correlation of CCN3 with anti-CCP and RF in RA patients. Serum CCN3 was positively correlated with anti-CCP (a) but not with RF (b) in RA patients. Spearman correlation analysis was conducted (anti-CCP: *r* = 0.500, *p* = 0.0009; RF: *r* = 0.276, *p* = 0.0801).

The inflammatory response during the development of RA often results with the change of a number of laboratory parameters. Although there was a strong positive correlation between CCN3 expression and disease activity score, most laboratory variables did not correlate with the serum CCN3 level (Table 2). It has been demonstrated that the anti-CCP antibody has a higher specifity for RA compared to RF. Among those laboratory parameters, the level of RA-specific autoantibody anti-CCP but not RF was positively correlated with the CCN3 level (anti-CCP: *r* = 0.500, *p* = 0.0009; RF: *r* = 0.276, *p* = 0.0801; Figures 3(a) and 3(b)).

### 3.5. The Relationship between the Expression of CCN3 and IL-6 in RA Patients

**Figure 4:**
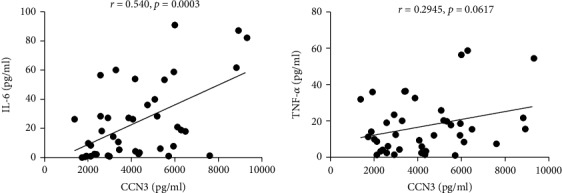
The correlation of CCN3 with inflammatory cytokines in RA patients. The ELISA was used to detect inflammatory cytokines from RA patients' sera. The determination of linear relationship between CCN3 and IL-6 or TNF-*α* was performed by the Spearman correlation coefficient. CCN3 is positively correlated with IL-6 (*r* = 0.4657; *p* = 0.0022).

As is well known, inflammatory cytokines such as IL-6 and TNF-*α* contribute greatly to the pathophysiological process of RA [22]. To explore the effect of CCN3 on the inflammatory cytokine production, the relationships between CCN3 and IL-6 or TNF-*α* were examined. A positive correlation was observed between CCN3 and IL-6 (*r* = 0.4657, *p* = 0.0022), but not between CCN3 and TNF-*α* (*r* = 0.2247, *p* = 0.1587) in RA patients (Figure 4). In consistency with this, IL-6 was also positively correlated with RA disease activity DAS28 (ESR) and DAS28 (CRP), suggesting that both CCN3 and IL-6 may serve as a biomarker for inflammation and disease activity in RA (Tables 3 and 4).

## 4. Discussion

In the present study, a significantly increased serum level of CCN3 was observed in RA patients, and the CCN3 expression was positively correlated with DAS28 (ESR), DAS28 (CRP), and anti-CCP antibody, which are critical clinical and laboratory parameters in RA. Furthermore, we observed a strong correlation between serum CCN3 and IL-6 level in RA patients. IL-6 is a common cytokine that plays a key role in the development of RA. These data suggest that CCN3 might be involved in the development of RA through regulating the inflammatory response.

As a member of matricellular proteins, CCN3 has recently received attention owing to its role in angiogenesis and fibrosis [23]. Treatment with recombinant CCN3 results in neovascularization rat corneas, an angiogenetic effect that might be related to its binding to integrins such as *α*v*β*5 [24, 25]. In renal fibrosis, CCN3 suppresses TGF-*β*1-induced extracellular matrix accumulation [26]. In addition, the impact of CCN3 was also observed in systemic scleroderma, an autoimmune rheumatic disease characterized by excessive production and accumulation of collagen in the skin, small arteries, and internal organs [27]. RA is typically characterized by sustained inflammatory synovitis. Previous studies showed that CCN3 was upregulated in RA synovial samples and osteoarthritis [14]. In the present study, we also found the CCN3 expression in the synovial samples from RA and osteoarthritis patients and CCN3 was much higher in the synovial tissue from RA compared to that in OA. Furthermore, the serum level of CCN3 was markedly increased in RA patients when compared with healthy controls.

Recently, the immunoregulatory role of CCN3 has become a rising concern. It has been demonstrated that blockade of CCN3 activity inhibits cell inflammation and apoptosis through downstream TGF-*β*/p-Smad and NF-*κ*B pathway in human alveolar epithelial cells [28]. Furthermore, recent studies also showed that CCN3 was a novel adipokine, and it had emerged as a potential metabolic regulator in type 2 diabetes [29]. In this study, we also found a positive correlation between CCN3 and disease activity score in RA patients, suggesting that CCN3 may play a critical role in the development of RA. In contrast, it has been shown that CCN3 exhibits a protective role in osteoarthritis and disruption of CCN3 in aged male mice results in osteoarthritis-like disease [30]. In addition, CCN3 is identified as a protective factor in cartilage by inhibiting the PI3K/AKT/mTOR pathway, alleviating the deterioration of osteoarthritis [31]. Therefore, CCN3 may play distinct roles in RA and osteoarthritis, two common types of chronic arthritis. As a chronic and systemic autoimmune disease, the inflammatory processes of RA involve immune cells, cytokines, chemokines, proteases, and matrix metalloproteinases (MMPs). In contrast, osteoarthritis (OA), an age-related degenerative joint disease, is pathologically characterized by articular cartilage degeneration. Infiltration of immune cells, especially T cells and macrophages in the synovial membrane of RA patients, leads to fibroblast expansion, osteoclast stimulation, and eventually tissue remodeling [32]. In consistency with this, CCN3 is recently identified as a Treg-derived mediator and plays a role in remyelination in multiple sclerosis [13].

In addition to T cells, macrophages also play a key role in the inflammation of RA. CCN3 is also associated with macrophage polarization into M1 (proinflammatory) and M2 (anti-inflammatory). In atherosclerosis, CCN3 inhibits macrophage foam cell formation [33]. Interestingly, the absence of CCN3 gene was associated with a change in macrophage profile (M1-like to M2-like) and proinflammatory cytokine/chemokine expression in adipose tissues. Macrophage is the main producer of TNF-*α* and IL-6. In our study, a positive correlation between CCN3 and inflammatory cytokine IL-6 was observed. There was also a trend of positive correlation between CCN3 and TNF-*α*, although it was not statistically significant. Besides, the CCN3 expression is also positively correlated with the anti-CCP antibody, which has been shown to promote macrophage polarization into M1 subset. These data show that CCN3 plays an important role in inflammation by regulating macrophage polarization and cytokines, resulting in a deleterious role in joint tissue destruction.

In summary, these results suggest that the serum CCN3 could be a sensitive marker for disease activity in RA patients. In addition, CCN3 was associated with inflammatory cytokines and anti-CCP antibody in RA, suggesting that the CCN3 may play a critical role in the pathogenesis of RA. Of course, further studies are required to explore the specific regulatory mechanism and the pathogenic role of CCN3 in RA.

## Figures and Tables

**Table 1:** Baseline characteristics of participants in this study.

Characteristics	RA patients	Healthy controls
Total (female/male)	41 (36/5)	45 (37/8)
Age, mean (SD) (year)	51.7 (16.3)	49.6 (12.1)
Duration of disease, mean (SD) (year)	6.2 (5.6)	—
DAS28 (CRP), mean (SD)	4.4 (1.6)	—
DAS28 (ESR), mean (SD)	4.7 (1.7)	—
CRP (mg/l), mean (SD)	57.5 (61.2)^∗∗∗^	4.9 (3.8)
ESR (mm/h), mean (SD)	49.9 (31.0)^∗∗∗^	11.3 (9.6)
RF (IU/ml), mean (SD)	580.2 (950.2)^∗∗∗^	16.2 (10.9)
Anti-CCP (RU/ml), mean (SD)	66.9 (63.8)^∗∗∗^	12.4 (8.1)
IgG (g/l), mean (SD)	14.5 (3.4)	13.8 (6.4)
IgA (g/l), mean (SD)	3.3 (1.3)	3.0 (1.7)
IgM (g/l), mean (SD)	1.5 (0.7)	1.1 (2.0)
WBC (10^9^/l), mean (SD)	7.8 (2.4)	6.9 (4.2)
Lym (10^9^/l), mean (SD)	1.8 (0.6)	1.9 (1.1)
PLT (10^9^/l), mean (SD)	347.9 (102.3)^∗∗^	196.3 (85.3)
Hb (g/l), mean (SD)	111.2 (14.6)^∗^	145.5 (21.8)

CRP: C-reactive protein; ESR: erythrocyte sedimentation rate; RF: rheumatoid factor; anti-CCP: anti-cyclic citrullinated peptide; Ig: immunoglobulin; WBC: white blood cell; Lym: lymphocytes; PLT: platelet; Hb: hemoglobin. ∗ indicates RA vs. healthy controls (HC), *p* < 0.05. ∗∗ indicates RA vs. HC, *p* < 0.01. ∗∗∗ indicates RA vs. HC, *p* < 0.001.

**Table 2:** Correlation analysis between CCN3 level and laboratory variables in patients with RA.

Characteristics	WBC	Lym	Hb	PLT	IgG	IgA	IgM
*r*	0.031	0.158	0.104	0.044	0.045	0.156	0.242
*p* value	0.859	0.363	0.553	0.803	0.812	0.411	0.198

Spearman's correlation analysis was used to calculate significance.

**Table 3:** Correlation analysis between DAS28 (ESR) and laboratory variables in RA patients.

Characteristics	WBC	Lym	Hb	PLT	IgG	IgA	IgM	IL-6	TNF-*α*	CCN3
*r*	0.142	-0.141	-0.026	0.128	0.098	0.410	0.454	0.481	0.205	0.483
*p* value	0.413	0.418	0.879	0.463	0.604	0.024^∗^	0.011^∗^	0.001^∗∗^	0.198	0.001^∗∗^

Spearman's correlation analysis was used to calculate significance. ∗ indicates *p* < 0.05; ∗∗ indicates *p* < 0.01.

**Table 4:** Correlation analysis between DAS28 (CRP) and laboratory variables in RA patients.

Characteristics	WBC	Lym	Hb	PLT	IgG	IgA	IgM	IL-6	TNF-*α*	CCN3
*r*	0.089	-0.140	0.045	0.115	0.050	0.430	0.586	0.427	0.224	0.487
*p* value	0.610	0.420	0.793	0.510	0.790	0.017^∗^	0.001^∗∗^	0.005^∗∗^	0.158	0.001^∗∗^

Spearman's correlation analysis was used to calculate significance. ∗ indicates *p* < 0.05; ∗∗ indicates *p* < 0.01.

## Data Availability

The data used to support the findings of this study are available from the corresponding author upon request.
